# Immunotherapy with *Pru p 3* for food allergy to peach and non-specific lipid transfer protein: a systematic review

**DOI:** 10.1186/s12948-023-00184-5

**Published:** 2023-05-31

**Authors:** Carlo Maria Rossi, Marco Vincenzo Lenti, Stefania Merli, Amelia Licari, Gian Luigi Marseglia, Antonio Di Sabatino

**Affiliations:** 1 Department of Internal Medicine and Medical Therapeutics, University of Pavia, Pavia, Italy; 2 First Department of Internal Medicine, Fondazione IRCCS San Matteo, Pavia, Italy; 3 Department of Clinical, Surgical, Diagnostic and Pediatric Sciences, University of Pavia, Pavia, Italy; 4 Pediatric Clinic, Fondazione IRCCS Policlinico San Matteo, Pavia, Italy; 5 Clinica Medica I, Fondazione IRCCS Policlinico San Matteo, Università Di Pavia, Viale Golgi 19, 27100 Pavia, Italy

**Keywords:** Diet, Food allergy, Immunotherapy, Non-specific lipid transfer protein, Peach

## Abstract

**Background:**

Non-specific lipid-transfer protein (nsLTP) is a pan-allergen in the plant world, and a cause of significant concern as food allergen in the Mediterranean area, due to its general heat- and acid-resistance and hence the risk of severe allergic reactions. *Pru p 3*, the peach nsLTP, is considered the primary sensitizer to this allergen family and this allergy is usually persistent. Allergen-free diet and acute treatment of manifestations are the main recognized management goals in food allergy.

**Main text:**

The role of immunotherapy for treating food allergy in adult patients is controversial, but immunotherapy for *Pru p 3* could potentially represent a relevant therapeutic strategy. We systematically searched databases for studies assessing the role of immunotherapy *Pru p 3* in food allergy. Overall, nine studies were included. Immunotherapy with *Pru p 3* appears to be effective and with a good safety profile in both peach and LTP allergy for some foods, such as peanut, in both RCT and real-life studies.

**Conclusions:**

Immunotherapy with *Pru p 3* is a possible treatment option for food allergy to the peach LTP in the Mediterranean area, although at present have not reached routinary clinical practice. Larger studies are needed to confirm these findings and identify predictive biomarkers.

## Background

Immunoglobulin E (IgE) food allergy is a specific immune-mediated adverse reaction to food allergens, representing a major health problem worldwide due to its steadily increasing prevalence, affecting up to 8% of children and 3% of adults in Western countries [1]. Particularly for some foods, such as peanuts, tree nuts, and crustaceans, allergy is usually persistent, whereas allergy to some others, such as milk, egg, and wheat tends to resolve with ageing [2, 3].

**Fig. 1:**
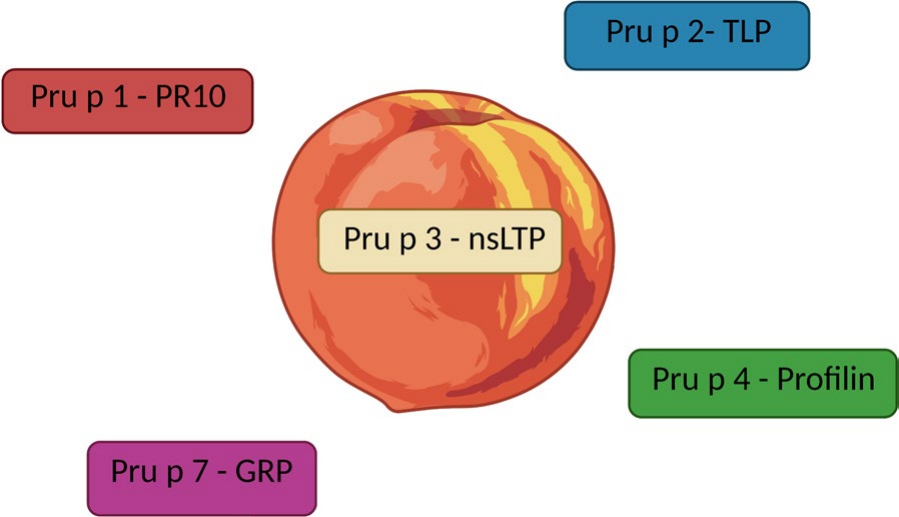
Peach main allergens, their physical–chemical characteristrics and associated clinical manifestations. *Pru p 1*, is a PR-10, a thermo- and acid-labile allergen; it is usually responsibile for mild reactions confined to the oropharynx. *Pru p 3*, a nsLTP is present only in the peel and is a thermo- and acid-resistant allergen, potentially responsibile for severe reactions. *Pru p 4* is the peach profillin, an acid-labile panallergen; it is usually responsibile for mild reactions confined to the oropharynx. *Pru p 7* a gibberlin-regulated protein, (GRP), is a thermo- and acid-resistant protein, which is present only in the pulp and potentially responsibile from severe reactions especially in Japan. *GRP* gibberlin-regulated protein, *PR-10* pathogenesis-related protein 10. Created with BioRender.com

Non-specific lipid-transfer protein (nsLTP) is a pan-allergen in the plant world, mainly present in the skin of fruits, and a cause of significant concern as food allergen in the Mediterranean area, due to its general heat- and acid- resistance and hence the risk of severe reactions, and due to its cross-reactivity among related or unrelated botanical species (Fig. 1). *Pru p 3*, the peach nsLTP, is generally considered the primary sensitizer to this allergen family, containing most of the relevant epitopes, at least in Southern Europe. Sensitization to nsLTP usually follows the gastrointestinal route, but cutaneous and respiratory route of sensitization have also been described. Allergy to nsLTP may be associated, despite a wide variability of clinical expression, with severe food allergic reactions and is persistent [4, 5].

Moreover, allergic reactions to nsLTP may by unpredictable, being influenced by aggravating factors, such as physical exercise, or non-steroidal anti-inflammatory drugs intake, especially in patients who are mono sensitized to this allergen, whereas a concomitant sensitization to pollens exerts a protective role. Besides, the spectrum of reactivity to different nsLTPs may widen with time, especially in patients with high basal level of IgE to *Pru p 3*. The sensitization/reactivity to multiple nsLTPs defines the so-called “LTP syndrome” [4, 5].

At present, the only recognized therapeutical strategies for food allergy according to guidelines are food avoidance and treatment of the acute manifestations in case of unintended allergen(s) ingestion [6, 7].

However, avoidance diets have been associated with significant limitations, including quality of life impairment and psychological, social, and economic burden on both patients and their families. Poor health-related quality of life correlates with the number of food allergies and is frequently present in patients with allergies to ubiquitous food/allergens [8]. These considerations also apply to allergy to fruit in general and nsLTP specifically, given its cross-reactivity.

More recently, allergen specific immunotherapy (AIT) has loomed over the therapeutical horizon as a promising strategy in the management of food allergy, with a product for peanut allergy being approved for oral immunotherapy (Palforzia, Aimmune Therapeutics) for children aged 4–17 and those becoming adult while on treatment [9, 10].

Indications for food immunotherapy, as issued by a European Academy of Allergy and Clinical Immunology (EAACI) position paper, include ineffectiveness of avoidance measures and poor quality of life [11]. AIT consists in the repeated allergen administration to modulate the immune response, including different routes such as the oral, the epi-cutaneous and the sublingual, i.e., OIT, EPIT and SLIT, respectively. It usually results in hypo-responsiveness during treatment, whereas desensitization or sustained hypo-responsiveness occur in a minority of patients [11].

Mechanisms of immunological modulation during AIT are elusive but include induction of regulatory T cells (Tregs) which may limit IgE production and enhance allergen-specific IgG (mainly IgG4) production and may inhibit mast cells and basophil activation [12].

Yepes-Nuñez JJ et al. [13] reviewed the efficacy and safety of immunotherapy (oral and sublingual) for food allergy to fruits in both children and adults, identifying one randomized clinical study that evaluated the effect of SLIT with *Pru p 3* on 56 adults by means of a double-blind placebo-controlled food challenge (DBPCFC) at baseline and after 6 months of treatment [14]. In the treatment group, evidence of desensitization (RR 1.16, 95% CI 0.49–2.74) was absent, whereas a significantly higher number of adverse effects was observed (RR 3.21, 95% CI 1.51–6.82). However, given the small sample size of the studies considered and the risk of bias, the authors declared that no definite conclusion can be made of the efficacy of immunotherapy for fruit allergy [13].

In light of the available studies (Table 1), until the release of the latest guidelines (2018) by the EAACI, food immunotherapy is recommended only for non-resolving allergies to cow’s milk, hen’s egg, and peanuts in the pediatric setting, whereas in adults immunotherapy for various foods, including peach, is not recommended, given the paucity and the low quality of available evidence [11].

**Table 1:** Human studies evaluating the efficacy and safety of LTP containing peach immunotherapy

First author, year, country	Study type/design	Study aim	Patient characteristics	Immunotherapy	Regimen	OFC	Immunological changes	Primary outcome/efficacy	Safety
Beitia, 2021 [15] (Spain)	Prospective, open label	To describe the outcome of peach SLIT in LTP syndrome	24 adults and five children treated with SLIT; 13 control individuals with LTP syndrome	SLIT with *Pru p 3*	-4-day build-up cluster schedule until reaching a dose of 50 μg of *Pru p 3* -Maintenance dose 12,5 μg/day of *Pru p 3* for 3 years	-No entry OFC -single-blind OFC with unpeeled peach (145 g), was performed after 1 year. Patients who failed the first test repeat OFC after 6 months and 1 year	–	-16/22 patients continuing therapy for 3 years passed to OFC after 1 year -5/22 patients passed OFC within 2 years -4/29 patients discontinued therapy for poor compliance - 7/13 patients of control group have reaction with other foods -The severity of symptoms in the control group increased significantly (p < 0.001)	-21/29 patients with OAS in the first weeks of SLIT, self-resolved -3/29 patients discontinued immunotherapy due to adverse events
Garcia-Gutierrez, 2021 [16] (Spain)	Case report	Try to stop the march of the LTP sensitization	A 46-year-old female with LTP syndrome reactive to hazelnut, mandarin, almond with contact dermatitis with peach	SLIT *with Pru p 3*	-Rush schedule in 1 day -Maintenance dose 10 μg/day of *Pru p 3* for 3 years	-No entry OFC - After 1-year single-blind OFC with peach (150 g) and mandarin 100 g) and aubergine (100 g)	After 2 years: -peach SPT decreased -total IgE increased -sIgE for *Pru p 3* decreased - peach LTP IgG4 increased	-150 g of peach (1–1.2 mg of *Pru p 3*) tolerated after 1 year -100 g of aubergine and mandarin tolerated after 1 year -No other symptoms with other foods	No adverse reactions
González Pérez, 2020 [17] (Spain)	Prospective, open label	To evaluate effectiveness of peach SLIT i in patients with other sensitizations	-18 patients (age 16–59 years) with LTP allergy including anaphylaxis -Presence of cofactors in 10 patients -17 patients with pollen rhinitis or asthma	SLIT with *Pru p 3*	-Rapid 2-day schedule, for 2 patients with OAS 4-day initiation schedule	-No entry OFC -After 1 year single-Blind OFC with peach	After 3 years: -*Pru p 3* IgE decreased -*Pru p 3* IgG4 increased	-At exit OFC tolerance in all patients	- 2 patients with OAS during induction -No adverse reactions during maintenance
Navarro, 2019 [18] (Spain),	Prospective, open label	To evaluate the effectiveness and safety of OIT with commercial peach juice	-24 patients (age 5–42 years) with a history of anaphylaxis in the previous 3 months -No control group	OIT with commercial fresh peach juice (*Pru p 3* concentration is 21,16 μg/ml)	-1/1000 starting dose to 5 ml at visit 17 -Maintenance dose 200 ml/day, for 4 days/week	-No entry OFC -Single-blind OFC at visit 18 with peach juice (cumulative dose 200 ml) or a fresh peach	-Peach SPT decreased -No difference in SPT with LTP	-19/24 patients passed the OFC after 3.6 months	-No severe reaction -7/24 patients with mild oral symptoms -2/24 patients presented urticaria associated to cofactors -5 withdrew from study
Moura, 2019 [19] (Portugal)	Retrospective, open label	To establish the safety of ultra-rush initiation protocol for peach SLIT	-15 patients (age 17–35 years) with a history of anaphylaxis with peach or other LTP foods -Presence of cofactors detected in 3 patients)	SLIT with *Pru p 3*	-Standard protocol in 4 days (n = 5) and ultra-rush-protocol in 2 days (n = 10). Standard protocol reaches a cumulative dose of 78 μg, ultra-rush protocol 47 μg -Maintenance dose 10 μg/day	-no OFC	After 1 year: -sIgE for peach decreased in 8 patients and increased in 3 patients of ultra-rush group -sIgE for *Pru p 3* decreased in 11 patients and increased in 3 patients of ultra-rush protocol and in 1 patient of standard protocol	-Ultra-rush-Protocol halved time to reach maintenance dose	-100% of patient in both groups with OAS during build-up phase -No systemic reaction -No increase of adverse reaction in ultra-rush-protocol -1 patient presented urticaria with exercise after ingestion of unpeeled apple after 1 year at the end of SLIT for 40 mouths
Gomez, 2017 [20] (Spain)	Prospective, open label	To evaluate peach and peanut desensitization and immunologic responses at 1 year after SLIT	-48 patients (30.8 years, 25–35) with systemic reactions to peach and/or peanut -36 patients treated with *Pru p 3* SLIT and 12 controls -12/36 patients with peanuts allergy	SLIT with *Pru p 3*	-4-day build-up cluster schedule -Maintenance dose 200 μg of *Pru p 3* for 1 year	-DBPCFC with 150 g of peach and 14 g of peanut was performed at T0 and after 12 month of SLIT -No DBPCFC in patients with 2 anaphylaxis episodes in the last 2 years	After 1 year: -SPT for peach and peanuts decreased in treated group -sIgE *Pru p 3* and *Ara h 9* decrease -sIgG4 for *Pru p 3* and *Ara h 9* increased -Basophil reactivity increased for *Pru p 3* and *Ara h 9*	-After 1 year increased threshold in OFC with peanut and peach in treated patients (p < 0.001) - 3/36 patients did not pass DBPCFC after 1 year -5 patients withdrew in the active group, in the placebo one	-25/36 with mild adverse reactions -In 91% of treated patients no reaction with peach after 1 year - No reaction after 1 years of *Pru p 3* SLIT in 7/12 patients with peanut allergy
Garcia, 2010 [21] (Spain)	RCT	-To evaluate the effect of peach SLIT to IgE levels to Rosaceae allergens, -To monitor new-Sensitization to Rosaceae or pollen	-56 peach allergic adult patients (18–65 years) randomized 2:1 to receive SLIT with peach or placebo for 6 months -21 patients with systemic reaction (mainly sensitized to *Pru p 3*) -Sensitization to *Mal d 1* in 18.5%, *Mal d 4* 24.1% at baseline	SLIT with peach peel extract (*Pru p 3* quantified). *Pru p 1* and profilin also present	Same protocol as adopted in Fernandez-Rivas (14	DBPCFC with lyophilized peach peelings at T0 and after 6 months of SLIT (cumulative dose of *Pru p 3* is 3249 μg, about 1,5 peaches)	- Peach SPT decreased in active group -sIgE for *Pru p 3* increased in both groups	-IgE to *Pru p 3* increased at 1 month in both active and placebo group but remained stable only in active group at 6 months -IgE to other allergens unchanged; no new sensitization detected -SPT to peach decreased in active group and were lower than controls at 6 months	/
Pereira, 2009 [22] (Spain)	Case report	To evaluate efficacy and safety of SLIT	-A 40 year-old female with LTP syndrome	SLIT with *Pru p 3* (concentraction of 40 μg/ml)	-Ultra-rush -protocol in 1 day -Maintenance dose of 10 μg/day for 5 days/week for 1 year	-DBPCFC with peach at T0 and after 4, 8 and 12 months after starting SLIT -OFC performed with 150 g of peach	-*Pru p 3* SPT decreased -No change for *Pru p 3* IgE, IgG, IgG1 and IgG4	- Negative OFC after 4 months - After 3 months of non-diet restriction for vegetable, only nuts and pepper were excluded by diet	- OAS during build-up -No adverse reaction during maintenance doses
Fernandez-Rivas, 2009 [14] (Spain)	-RCT	-To evaluate the change in response to DBPCFC with peach	-56 adults (18–65 years) with peach allergy randomized 2:1 to receive SLIT with peach or placebo for 6 months	SLIT with *Pru p 3*	-rush- protocol (until 50 µg of *Pru p 3*) -Maintenance dose of 10 μg/day for 3 days at week for 6 months	DBPCFC with lyophilized peach peelings at T0 and after 6 months of SLIT (cumulative dose of *Pru p 3* is 3249 μg, about 1,5 peaches)	-*Pru p 3* SPT decreased in treatment patients -*Pru p 3* sIgE increased in active and placebo arms -*Pru p 3* IgG4 increased in active arm	-Active group tolerated a 3 to ninefold higher amount of peach, presented a 5.3 times decrease in *Pru p 3* SPT and an IgG4 increase	-in 13/44 patients and 12 control side effects (OAS, gastrointestinal symptoms, urticaria and itching, rhinitis) -No serious effect reported

*DBPCFC* double blind placebo controlled food challenge, *DCs* dendritic cells, *LPS* lipopolysaccharides, *LTP* lipid transfer protein, *NSAID* non-steroidal anti-inflammatory drug, *OAS* oral allergy syndrome, *OFC* oral food challenge, *OIT* oral immunotherapy, *pbp* prick by prick, *RCT* randomized controlled trial, *SLIT* sublingual immunotherapy, *SPT* skin prick test, *T* time

However, more recently, additional studies with different inclusion criteria (comprising pediatric patients, patients with LTP syndrome and thus not only allergic to peach) and design (different aims, protocol of desensitization, follow-up, etc.…) have further examined this issue*.* Herein we revise in a systematic fashion the literature on the efficacy and safety of immunotherapy with *Pru p 3* nsLTP on peach and/or nsLTP allergy with a focus on clinical and therapeutical implications.

## Methods

**Fig. 2:**
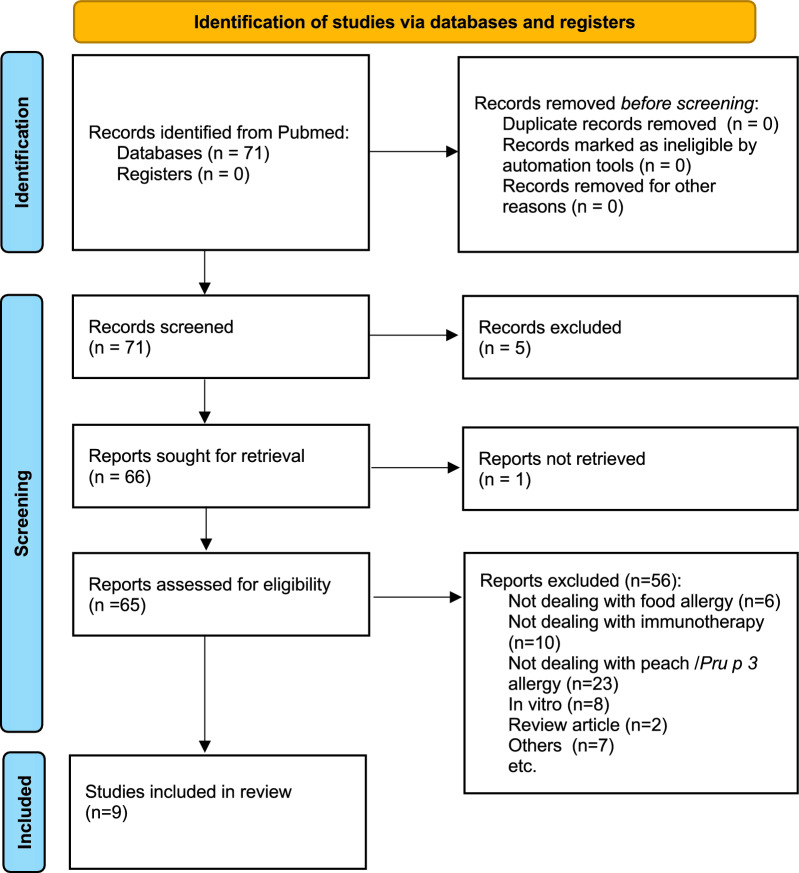
Flow diagram describing the searching of databases and selection of studies

In September 2022 we performed a Medline search (Pubmed, The Cochrane Database, and ClinicalTrials.gov) with the terms, “allerg*” “immunotherapy”, “EPIT”, “OIT”, “SLIT” and “*Pru p 3*”, “peach” and “(ns)LTP”. All types of human studies, both in children and adults, in English language, since database inception, were considered. Thereafter, a systematic search with the MeSH terms “allerg*” “immunotherapy” and “peach” was performed by using the Ryyan software (https://rayyan.ai). By applying these criteria, 71 studies were retrieved and reviewed by the study coordinators (CMR, SM) for assessing eligibility for inclusion. Reasons for exclusion were as follow; a) in vitro studies, not dealing with humans; b) studies not dealing with food allergy; c) studies not dealing with immunotherapy; d) non-original articles; e) studies not dealing with peach allergy. Case reports or series were included. The flow diagram reporting the study selection is shown in Fig. 2. Eventually, nine studies were included in the review (Table 1) [14–23]. The general considerations, efficacy, safety, and limitations of immunotherapy will be discussed.

### General considerations regarding human studies

The studies retrieved (Table 1) are heterogenous in terms of study design and population included; however, some general considerations can be made. Except for one [19], all studies were performed in Spain. The majority of enrolled patients were young female and a frequent co-sensitization to pollens was present [16, 17].

Most studies were open labelled, except two including randomization [14, 16]. Four studies also included pediatric patients [15–19]. Of note, some studies included patients with anaphylaxis [15, 17, 19] which is usually an exclusion criterion in clinical trials. For the same reason, most studies did not select patients on the basis of an oral food challenge (OFC) with peach but rather on clinical grounds, together with evidence of *Pru p 3* sensitization.

As far as administration route is concerned, all studies dealt with SLIT adopting a standardized commercial extract enriched in *Pru p 3*, except the one by Navarro, which evaluated the oral route for immunotherapy with commercial peach juice containing *Pru p 3* [18]. No studies dealing with the epicutaneous route of administration were found.

Most studies adopted a protocol comprising build-up phase of several days, usually four, but rush and ultra-rush protocols, carried out in 2 and 1 day respectively, were also used [^14^,^16^, ^17^, ^18^, ^19^, ^20^, ^21^].

The maintenance phase duration varies across studies from 6 months to 3 years, and the evaluation of tolerance varied across studies, comprising either single-blind [15, 17, 18] or double-blind oral food challenges [14, 16, 22].

### Efficacy

Efficacy was assessed by means of an OFC -percentage of passed tests- and additionally by measuring the wheal diameter of the skin prick test with *Pru p 3* or peach-with a decrease in this parameter being interpreted as a sign of response to the treatment.

Overall, the efficacy of SLIT with *Pru p 3* in inducing desensitization to this allergen is high, ranging from 72 to 100%, as assessed by an OFC (usually performed at 1 year of treatment). Interestingly, in the study by Gomez, in the minority of patients (3/36) maintaining reactivity to peach an increase in the threshold at OFC was observed [20].

The beneficial effect of immunotherapy was also present in the study by Navarro et al. [18], which evaluated the effect of OIT with *Pru p p 3* at 3.6 months with a rate of passed OFC of 79%. Moreover, in this study a commercial juice containing *Pru p 3*, rather than a standardized commercial extract, was used. This finding may also have practical implications due to the much lower costs of this mean as compared to commercial extract products.

In studies evaluating the wheal diameter of the skin prick test to *Pru p 3* as a measure of clinical efficacy, a decrease was observed [16, 18, 20].

Moreover, the clinical efficacy of immunotherapy with *Pru p 3* was substantiated by the evidence of concurrent immunological changes after the onset of treatment. More precisely, a decrease in titer of IgE levels to *Pru p 3* paralleled by an increase in IgG4 to *Pru p 3* were observed. On the contrary, in the only study evaluating basophil activation test as an immunological parameter, an unexpected increase in reactivity, *i.e.* occurring despite clinical response, was observed [20]. This finding was explained by the authors, among other theories, by the frequent contact of patients with the pan-allergen nsLTP in pollen- or food- sources, or possibly by the reduced number of laboratory determinations of this parameter, so that the observation reflects only a transitory phenomenon.

Of note, not only AIT with *Pru p 3* improves tolerance to peach but also it appears to exert beneficial effects also on allergy to other nsLTP-containing foods, such as peanut, the most studied, and hazelnut.

More precisely, the study by Beitia et al. [15] assessed whether a SLIT with *Pru p 3* could modulate the reactivity to nsLTP-containing food in patients with the LTP syndrome in a real-life setting. Patients enrolled (29, five children) were mainly allergic to the *Rosaceae* family, including a significant proportion of patients with cases of severe anaphylaxis (65.6%) to multiple fruits and vegetables (including peanut and nuts in 72% of the cases) in the previous year. LTP syndrome was diagnosed on a clinical ground. A positive OFC to peach was not an inclusion criterion. Patients allergic to peanut were sensitized to *Ara h 9*, the peanut nsLTP. Twenty-two patients completed the 3-year study, while seven patients discontinued the trial due to poor compliance or adverse reactions which abated with treatment interruption. The proportion of patients passing an OFC with unpeeled peach was 75% at 1 year and 95% at 2 years. Moreover, among the 16 patients allergic to peanuts 69% passed an OFC with peanut. At the end of the study period 20/21 patients had a normal diet. On the contrary, in the control group (13) half of the patients presented a reaction with new foods after accidental exposure, with an increase in the severity of symptoms as assessed by the Sampson criteria, as compared to baseline. Moreover, the number of avoided family food plant foods increased during a median period of follow-up of 3.7 years. Therefore, these patients needed to maintain a diet restriction.

In the study by Gomez et al. [20] an increase in the threshold during an OFC with peanut paralleled by a decrease of the wheal in the skin prick test to peanut was observed in the entire subset of patients allergic to this food (n = 12).

The fine mechanisms underpinning the beneficial effects of *Pru p 3* immunotherapy in preventing LTP syndrome progression -from peach to other less botanically-related food- are still elusive. However, some theoretical considerations and clinical findings are worth mentioning. More precisely, AIT has been found to prevent epitope spreading in human studies of house dust mite sensitization and cedar pollen allergy [23, 24]. Along with this concept, given that *Pru p 3* among all nsLTP contain most immunogenic epitopes, immunotherapy strategies using peach nsLTP could prevent epitope spreading and antibody affinity maturation, hence reducing the development of allergies to new nsLTP containing food. Alternatively, the development of blocking antibodies to *Pru p 3* could prevent the recognition of the same epitope in other nsLTP containing food. To further strengthen these concepts, following peach avoidance, new allergies to nsLTP containing food, especially peanut and hazelnut, may arise, even though patients sensitized, but not allergic to nsLTP other than peach, are allowed to keep consuming them, as found in a monocentric Italian prospective study of patients allergic to *Pru p 3* [25].

Taken together, these results suggest that SLIT with *Pru p 3* could be associated also with increased tolerance to other *Rosaceae* foods other than peach, with comparable desensitization rates to immunotherapy for peach also for other foods such as for peanut.

Finally, sustained hypo-responsiveness, which refers to the absence of reactivity to an allergen after the end of therapy and may correspond to allergy remission, was generally not investigated across the studies. In the study by Moura et al. [19], it was reported that a patient presented urticaria with an unpeeled apple at 1 year after the completion of SLIT for 40 months.

### Safety

Safety of immunotherapy is of great importance during all phases of treatment, *i.e.,* from induction and build-up to maintenance. This requisite is particularly important in the case of immunotherapy with *Pru p 3,* given its allergenic characteristics being potentially responsible for anaphylaxis.

A good safety profile of the immunotherapy was consistently observed among studies with both sublingual and oral routes of immunotherapy. Despite a significant frequency of adverse effects, in the study by Beitia [15], up to 72% of patients reported adverse reactions that were characterized by mild symptoms, which were localized to the oropharynx and occurred mainly in the first weeks of treatment, during induction or in the build-up phase, usually transient and responsive to anti-histamines.

Only one case of an oral allergic syndrome refractory to antihistamines was reported [19]. The patient who initially underwent a standard protocol was then switched to an ultra-rush protocol which was well tolerated.

Systemic symptoms confined to the skin (urticaria) were reported in only two patients among the 24 undergoing oral immunotherapy in the study by Navarro et al. [18] However, the presence of co-factors was deemed an explaining factor.

No cases of overt eosinophilic esophagitis were reported. In the study by Beitia et al. [15] a patient withdrew from the study for dysphagia, but no additional data were given, particularly as to whether the patient underwent an endoscopic examination of the upper gastrointestinal tract.

### Limitations of the available studies

Despite the evidence of clinical efficacy of the treatment, some limitations of the available studies should be highlighted. Some limitations refer to the methodology, while others to the study populations.

First, an OFC, ideally a DBPCFC should be performed, both at baseline and after completion of oral immunotherapy, to identify the threshold of reactivity (for baseline food challenge) and measure the response to the treatment, if any, and any improvement in the amount of the food allergen tolerated (for final food challenge). An entry OFC was not performed in some studies [15, 16, 18] mainly due to safety reasons, such as an history of anaphylaxis often with several episodes [15, 17, 18].

Second, the overall small sample size, comprising of mainly young females, and the limited geographical areas under study, almost exclusively Spain, hamper the generalizability of the results, so that larger studies are awaited to ascertain the efficacy of immunotherapy with *Pru p3*.

Finally, sustained hypo-responsiveness, an important measure of efficacy, has not been evaluated across studies. This measurement would reflect the possible restoration of oral tolerance to the allergen. Actually, this is even more relevant when considering that there are no predictive biomarkers of response in clinical practice.

## Conclusion

Immunotherapy with *Pru p 3* could be a relevant therapeutic option, and biomarkers could aid in identifying patients who are fit for the treatment. To minimize side effects, immunotherapy adopting allergoids, chemically modifies allergens with reduced allergenicity but unaltered immunogenicity, and a combination of biological agents targeting type 2 inflammation and immunotherapy, could be adopted. Costs are also a concern, since immunotherapy is expensive and, in many countries, not reimbursed by the health care system. Predictive biomarkers of response and the use of standardized natural extracts may allow a proper patient selection and may reduce the costs.

## Data Availability

Data are available in the medical literature.

## Abbreviations

AIT: Allergen immunotherapy
nsLTP: Non-specific lipid transfer protein
OIT: Oral immunotherapy
